# The Impact of Cognitive Style Diversity on Implicit Learning in Teams

**DOI:** 10.3389/fpsyg.2019.00112

**Published:** 2019-02-07

**Authors:** Ishani Aggarwal, Anita Williams Woolley, Christopher F. Chabris, Thomas W. Malone

**Affiliations:** ^1^Brazilian School of Public and Business Administration, FGV, Rio de Janeiro, Brazil; ^2^Tepper School of Business, Carnegie Mellon University, Pittsburgh, PA, United States; ^3^Geisinger Health System, Lewisburg, PA, United States; ^4^Sloan School of Management, Massachusetts Institute of Technology, Cambridge, MA, United States

**Keywords:** teams, team learning, implicit coordination, collective intelligence, cognitive diversity

## Abstract

Organizations are increasingly looking for ways to reap the benefits of cognitive diversity for problem solving. A major unanswered question concerns the implications of cognitive diversity for longer-term outcomes such as team learning, with its broader effects on organizational learning and productivity. We study how cognitive style diversity in teams—or diversity in the way that team members encode, organize and process information—indirectly influences team learning through collective intelligence, or the general ability of a team to work together across a wide array of tasks. Synthesizing several perspectives, we predict and find that cognitive style diversity has a curvilinear—inverted U-shaped—relationship with collective intelligence. Collective intelligence is further positively related to the rate at which teams learn, and is a mechanism guiding the indirect relationship between cognitive style diversity and team learning. We test the predictions in 98 teams using ten rounds of the minimum-effort tacit coordination game. Overall, this research advances our understanding of the implications of cognitive diversity for organizations and why some teams demonstrate high levels of team learning in dynamic situations while others do not.

## Introduction

Organizations are increasingly seeking the benefits of diversity, particularly the cognitive diversity that can enable the synthesis of different knowledge bases, perspectives, and opinions necessary to solve difficult problems (Uzzi et al., 2013). While the advantages of cognitive diversity for short-term problem solving and innovation have been demonstrated (Hong and Page, 2001; Ellemers and Rink, 2016), the impact of such diversity for longer-term outcomes, such as team learning, are less clear. Research looking at team learning captures the way in which teams change as a function of experience and can manifest itself in changes in processes, cognitions, routines, or performance, and has major implications for sustained organizational outcomes (Fiol and Lyles, 1985; Argote et al., 2001; Argote and Miron-Spektor, 2011; Kush et al., 2012). It furthers our understanding of teams as complex, adaptive, dynamic systems that are embedded in constantly changing contexts (Ilgen, 1999; McGrath Joseph et al., 2000).

Despite the theoretical logic, the direct link between team diversity and team outcomes is not supported by conclusive empirical evidence, and a deeper study of the mechanisms through which team diversity is likely to influence team outcomes has been called for (van der Vegt and Bunderson, 2005; Hülsheger et al., 2009; Jackson and Joshi, 2011). Accordingly, we identify and study an important mechanism that is likely to mediate the impact of cognitive diversity on team learning, i.e., collective intelligence. Recent research shows that some teams have a greater general ability to work together across a wide range of task types, referred to as collective intelligence (Woolley et al., 2010). While a certain amount of cognitive diversity may enhance collective intelligence by supplying the necessary cognitive inputs and differentiators for task work, too much diversity induces high coordination costs as members with different perspectives have a hard time understanding each other (Steiner, 1972; Cannon-Bowers et al., 1993; Cronin and Weingart, 2007; Mello and Rentsch, 2015; Aggarwal and Woolley, 2018). We further predict this hurts collective intelligence in the short run, as well as team learning over the long run (Van der Vegt and Bunderson, 2005).

Our key premise, thus, is that cognitive style diversity, by providing the building blocks to the team through composition, will impact team learning indirectly through its influence on the team’s collective intelligence. We combine existing theory to theorize the relationship among team cognitive diversity, collective intelligence and team learning, and specifically predict that (a) cognitive style diversity has a curvilinear (inverted U-shaped) relationship with collective intelligence, with high levels of cognitive diversity disrupting collective intelligence; (b) collective intelligence enhances the rate of learning of implicit coordination in teams; and (c) high levels of cognitive diversity impede learning of implicit coordination via the disruption of collective intelligence. We examine these relationships in the context of a laboratory study with 98 teams, finding that cognitive diversity maintains a non-monotonic, inverted-U shaped relationship with collective intelligence, and that collective intelligence is a mechanism whereby cognitive diversity impacts team learning. We discuss implications for both research and practice.

## Theoretical Background

### Team Cognitive Diversity

Teams can be diverse in many different ways, both in terms of observable characteristics such as race and gender as well less overtly observable differences in what members believe, know, or think (Harrison et al., 1998; Mannix and Neale, 2005). Most of the benefits of diversity stem from the cognitive inputs it can make available to teams; by influencing the activities of thinking, knowing, and processing information, the team’s cognitive inputs are likely to provide it with the essential building blocks to process information that is directly applicable to the tasks they encounter (Hong and Page, 2001; Phillips and Loyd, 2006; Woolley et al., 2008; Mello and Rentsch, 2015; Aggarwal and Woolley, 2018). While there are several cognitive dimensions that can inform a team’s cognitive diversity, one construct that has existed in the field of psychology for decades, but only recently explored in the context of teams, relates to cognitive styles. Cognitive styles are psychological dimensions that represent consistencies in how individuals acquire, organize and processes information, and are relatively permanent characteristics that are not necessarily associated with differences in intellectual abilities (Bartlett, 1932; Paivio, 1971; Richardson, 1977; Ausburn and Ausburn, 1978; Riding and Cheema, 1991; Armstrong and Priola, 2001; Kozhevnikov et al., 2014).

One cognitive style dimension that has been studied in the context of teamwork is the verbal-spatial-object-visualization dimension (Schilpzand, 2010; Aggarwal and Woolley, 2013, 2018). Existing research demonstrates that those strong in visualization rely primarily on imagery when attempting to perform cognitive tasks, whereas those strong in verbalization rely primarily on verbal analytical strategies. Furthermore, there are two different types of visualization; individuals strong in *object* visualization use holistic processing and perform better on tasks that require identifying global properties of shapes, whereas individuals strong in *spatial* visualization encode and process images analytically, part by part, using spatial relations to dissect, arrange and analyze the components (Bartlett, 1932; Paivio, 1971; Richardson, 1977; Kozhevnikov et al., 2005; Kozhevnikov et al., 2014).

These cognitive styles are globally relevant to a wide variety tasks, as compared to other types of cognitive styles which are more specific to decision-making (Schwartz et al., 2002) or problem-solving (Kirton and Kirton, 1994). They provide individuals with lenses through which they encode, process and organize information, and guide an individual’s information processing, decision making, and problem solving, as well as their proclivity for and performance in artistic and scientific pursuits (Kozhevnikov et al., 2005; Blazenkova et al., 2006, 2011; Chabris, 2007; Kozhevnikov, 2007; Blazhenkova and Kozhevnikov, 2010; Moskvina and Kozhevnikov, 2011; Kozhevnikov et al., 2013). Given that we are interested in the impact of cognitive diversity on collective intelligence, or a team’s general ability to perform together across a variety of tasks, we focus on these dimensions of cognitive diversity that reflect stable cognitive tendencies that are applicable across contexts (Streufert and Nogami, 1989; Hayes and Allinson, 1998).

### Team Collective Intelligence

A team’s collective intelligence is its general ability to work together across a wide range of tasks (Woolley et al., 2010). Many theorists have put forth that groups in organizations function as information processing systems (Hinsz et al., 1997). Weick and Roberts (1993) suggested that the key to understanding collective cognition is recognizing that “mind” is located in patterns of connections between individuals, rather than in entities or elements, and to understand group mind is to understand process. A collective intelligence perspective studies a team’s capacity to integrate all of its resources and processes, and be adaptive, enabling a consistent level of performance across different task contexts that often impose competing demands on teams.

Given the variety of problems teams face, there is likely to be a delicate balance between not having enough cognitive diversity and having too much for developing high levels of collective intelligence. Not having enough cognitive diversity means lacking some key cognitive resources to tackle the range of tasks the team faces (van Knippenberg and Schippers, 2007; Van Knippenberg and Mell, 2016). A lack of diversity will also mean that a team will lack differentiators and indicators of which member brings necessary knowledge and skills for the work, making it more difficult to use the skills the team possesses (Phillips and Loyd, 2006; Aggarwal and Woolley, 2018). At the same time, having too much cognitive diversity can lead to coordination costs that exceed the potential benefits of the team members’ contributions, where teams perform at lower levels than they are capable of performing given their team inputs (Steiner, 1972; Reagans and Zuckerman, 2001). We consider this balance when theorizing about the impact of cognitive diversity on collective intelligence, and we underscore the importance of this balance regardless of the conceptualization of cognitive diversity.

For example, when diversity is assumed to be indicative of a variety of cognitive resources, having a moderate level is most likely to benefit collective intelligence. This is because, on the one hand, as argued by the information-processing perspective, having an adequate range of cognitive style resources in the system is likely to give teams the necessary materials to deal with complexity in the environment when performing tasks that impose competing demands. This is also reiterated by the law of the requisite variety, which posits that variety within a system must be at least as great as the environmental variety against which it is attempting to regulate itself (Ashby, 1958). On the other hand, as argued by the shared cognition perspective, the fuller range of perspectives enabled by diversity also brings the possibility that members of the team may not understand each other (van Knippenberg and Schippers, 2007). In another conceptualization of cognitive diversity, where diversity is assumed to be indicative of cognitive distance or differences among team members on a cognitive attribute, having a moderate level of cognitive diversity is also likely to be most beneficial to collective intelligence. This is because, on the one hand, as argued by the signal-detection perspective, heterogeneity is likely to intensify the signals about the distribution and location of cognitive resources, and facilitate the reaching of an accurate understanding of how the skills are distributed among the team members (Aggarwal and Woolley, 2018). On the other hand, as argued by the shared cognition and representational gaps perspectives, when individuals are very different from each other, they have different mental representations or models, which increases the likelihood that team members will perceive the task differently, leading to gaps in their interpretations of taskwork (Cronin and Weingart, 2007). This is seen in existing research that has shown that cognitive style diversity facilitates the formation of transactive memory systems, or the understanding of how skills and expertise are distributed within the team, but at the same time hampers the formation of strategic consensus or being on the same page about the strategic priorities of the team (Aggarwal and Woolley, 2013, 2018).

Taking these arguments together leads us to expect that moderate levels of cognitive diversity will yield the highest levels of collective intelligence, as this is the point at which teams will be able to accrue the benefits of diversity while not yet experiencing the high costs. Accordingly, we hypothesize a non-monotonic, curvilinear (inverted U-shape) relationship between team cognitive style diversity and collective intelligence, such that the highest levels of collective intelligence are likely to emerge in teams with a moderate level of cognitive style diversity. This is in line with past research that has presented non- monotonic relationships pertaining to diversity (Earley and Mosakowski, 2000; Gibson and Vermeulen, 2003; Dahlin et al., 2005; van der Vegt and Bunderson, 2005). Accordingly, we predict:

Hypothesis 1: There will be a curvilinear—an inverted U-shaped—relationship between cognitive style diversity and collective intelligence.

### Team Learning: Implicit Coordination

Team learning can be defined as change in the team that occurs as a function of experience and can manifest itself in changes in processes, cognitions, routines, or performance (Fiol and Lyles, 1985; Argote et al., 2001; Argote and Miron-Spektor, 2011; Kush et al., 2012). Team learning has also been defined as a change in the group’s repertoire of potential behavior that occurs over time in group interaction (Wilson et al., 2007; Schippers et al., 2013). For example, as groups gain experience they may acquire information about how to use a new piece of technology more effectively, or how to coordinate their activities better (Argote, 1999, p. 100). Understanding how groups change as a function of experience is an important—though complex—undertaking, where it is important to be sensitive to a significant component of learning that is tacit in nature (Argote, 1999, p. 101). Accordingly, we undertake this task when studying team learning, and we focus on a tacit component i.e., the change in a team’s implicit coordination (or coordination based on subtle and dynamic cues) that occurs as a function of experience (Wittenbaum et al., 2002).

Coordination, which may be either implicit or explicit in nature, is one of the major problems in organizations that teams must face and solve in order to be effective (March and Simon, 1958; Georgopoulos, 1972; Okhuysen and Bechky, 2009). Explicit coordination requires that team members verbally communicate in order to articulate plans, define responsibilities, negotiate, and seek information to undertake common tasks. By contrast, implicit coordination requires the team to anticipate the actions and needs of their colleagues, and dynamically adjust their own behavior, *without* explicit communication (Salas and Cannon-Bowers, 2001; Wittenbaum et al., 2002; Espinosa et al., 2004; MacMillan et al., 2004; Rico et al., 2008). In a fast-paced and constantly changing environment, the success of a team often depends greatly on its ability to coordinate implicitly (Marques-Quinteiro et al., 2013). Hence, it has been argued that implicit coordination forms an important basis for teamwork in a variety of settings such as high-reliability organizations including hospitals (Argote, 1982; Valentine and Edmondson, 2014), airplanes (Weick and Roberts, 1993), emergency response (Xiao et al., 1996) and product development (Crowston and Kammerer, 2010), among others, where teams have to respond in real time and in ambiguous situations.

The ability to coordinate implicitly incorporates both anticipation and dynamic adjustment among team members and is critical to achieving adaptation and performance on complex tasks. For example, Entin and Serfaty (1999) found that teams in stressful and high-workload situations were more likely to perform well when members used implicit rather than explicit coordination strategies. Thus, improvement in implicit coordination can be critical to long-term effectiveness in a variety of environments.

Collective intelligence entails the ability to communicate and transfer information efficiently (Woolley et al., 2015; Chikersal et al., 2017), which we theorize are processes that should enable highly collectively intelligent teams to improve the rate at which they coordinate implicitly. Further, the demonstrated ability of a team to consistently perform well across a wide array of task domains reflects flexibility and hints at an underlying capacity of the team to adjust to changing demands of the task and synchronize its task strategies and use of member resources quickly, as opposed to rigidly patterned responses (Gorman and Cooke, 2011). This capacity to adapt to its environment in order to operate effectively is associated with its learning-related behaviors (van der Vegt and Bunderson, 2005).

This prediction parallels a long line of research connecting individual intelligence and individual learning, which shows that the more efficient information processing that is characteristic of highly intelligent individuals enables them to gain more information from a given amount of experience, learning more quickly (Deary, 2000; Finn et al., 2014). Additionally, the link has been studied at the organizational level (Levinthal and March, 1993), and learning is considered a central basis for intelligent and effective organizational action (March and Simon, 1958; Cyert and March, 1963; Hedberg et al., 1976; Levitt and March, 1988; Huber, 1991; Argote, 1999). Similarly, at the team level, we would expect that highly collectively intelligent teams would be superior at encoding, transferring and applying lessons from early experiences to later experiences, enabling team learning. Accordingly, we predict:

Hypothesis 2: Collective intelligence in teams will be positively correlated with teams’ rate of implicit learning.

Coordination losses are among the most detrimental costs associated with the use of teams (Steiner, 1972). And, as discussed above, high levels of diversity have been known to disrupt coordination in teams. When team members have similarity, rather than differences, in cognitive styles, they are more likely to accurately anticipate each other’s actions in the absence of verbal communication. Hence, even as teams gain experience, teams with high levels of cognitive style diversity will have a slower rate of improvement in their implicit coordination, as compared to teams that are more homogeneous. Further, the influence of team cognitive diversity on team learning is likely to occur via mechanisms that involve shared information processing. The processing of information in groups involves activities that occur within as well as among the minds of group members and represents the degree to which information, ideas, or cognitive processes are exchanged among the group members, giving rise to team processes and emergent states (Ickes and Gonzalez, 1994; Hinsz et al., 1997; Marks et al., 2001). As argued above, high levels of cognitive style diversity are likely to disrupt these information transfer processes, thus disrupting collective intelligence and, by extension, team learning. Accordingly, we expect that cognitive style diversity is likely to influence the rate at which teams learn to coordinate implicitly via collective intelligence. Specifically, having high levels of cognitive style diversity is expected to hurt the team’s collective intelligence, which is expected to benefit team learning. Thus, we predict:

Hypothesis 3: There will be an indirect relationship between cognitive style diversity and team implicit learning, mediated by collective intelligence.

## Study

### Methods

#### Participants

The sample consisted of 337 participants, randomly assigned to 98 teams of two to five participants each. The participants were recruited from the general public in the northeastern United States and paid for their participation. The mean age was 23.6 years; 53% were male. Subjects in this study consisted of a subset of the participants from a larger study (Woolley et al., 2010). This study was carried out in accordance with the recommendations of the guidelines of research involving human subjects, Office of Research Integrity and Compliance, Carnegie Mellon University. The protocol was approved by the Carnegie Mellon University’s Institutional Review Board. All subjects gave written informed consent.

#### Procedure and Task

After the participants filled out the consent form, they filled out survey measures of cognitive styles individually (see below). Subsequently, each team worked on the collective intelligence battery (Woolley et al., 2010) for up to 4 h. Following this, the teams participated in the minimum-effort tacit coordination game for approximately twenty-five minutes. Participants could earn up to $10 per member based on group performance in the minimum-effort game, on top of the $40 paid for the earlier portion of the study.

The minimum-effort tacit coordination game (Van Huyck et al., 1990) is used to explore the ability of groups to implicitly coordinate their strategy. The game involves multiple rounds of individual decision making in which the team gains or loses money as a result of the decisions made by team members who make their decisions simultaneously and without communication. The key to success is to coordinate action with the counterpart, which would be less challenging if the participants could talk to each other. The secure choice is to exert minimal effort which insures the minimum positive payoff. Exerting maximum effort creates the possibility of earning the maximum payoff, but carries the risk of a negative payoff (Deck and Nikiforakis, 2012).

**FIGURE 1:**
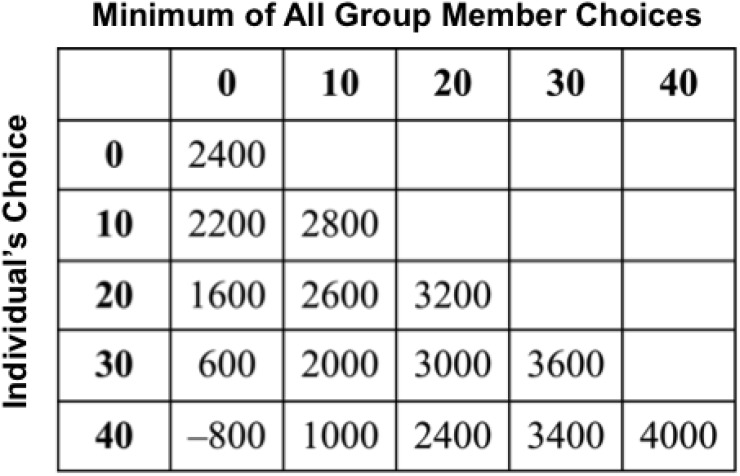
The payoff matrix of the minimum effort tacit coordination game.

This game was conducted in 10 rounds. In each round, each team member chose a number 0, 10, 20, 30, or 40. At the end of the round, each member received points as determined by the payoff matrix in Figure 1, which took into account the member’s own choice and the minimum of all member choices on that round. The maximum payoff for an individual, 4,000 points, occurs when the individual chooses 40, and the minimum choice in the team is 40 (i.e., everyone else in the group also chooses 40, indicating that their strategy is coordinated). Choosing 40, however, is also the riskiest strategy because, if the group’s minimum is 0, then any member who chooses 40 would lose 800 points. The safest choice for the individual is to choose 0, which would make the group minimum also 0, and result in a suboptimal gain of 2,400 points for the individual. Hence, teams earn more if they coordinate by making the same choices, with the optimal choice being for all members to choose 40. By contrast, a lack of coordination (i.e., making different choices) leads to poorer outcomes (Figure 1). Individuals do not have knowledge of the choices of others before making a decision. They are informed about the group’s minimum for the round at the end of each of the ten rounds, after which they have 60 s to make their choice for the next round. This game differs from the standard prisoner’s dilemma game in that teams are rewarded for coordinating rather than for competing.

#### Measures

*Cognitive Style*. The object-spatial imagery and verbal questionnaire (OSIVQ) (Blazhenkova and Kozhevnikov, 2009) was used to measure cognitive styles. For each participant, the 15 item ratings from each factor were averaged to create object, spatial and verbal scores (*M* = 3.46, *SD* = 0.51 object scale, *M* = 3.07, *SD* = 0.60, spatial scale, *M* = 3.17, *SD* = 0.47 verbal scale). The Internal reliability (Cronbach’s α) for the object scale was 0.81, the spatial scale 0.85, and the verbal scale 0.76. *Cognitive style diversity* was captured as the sum of the within-team standard deviation in each cognitive style. *Cognitive style level* was used as a control variable and captured as ${\left(\sqrt{\mathrm{OVmean}}+\sqrt{\mathrm{SVmean}}+\sqrt{\mathrm{Vmean}}\right)}^{2}$ which is similar to, but a more robust measure than, the sum of the means of the three cognitive styles, particularly when team size varies (Aggarwal and Woolley, 2018).

*Collective Intelligence* was measured as the factor capturing the team’s performance across a battery of tasks, as described in Woolley et al. (2010). In this battery, tasks are sampled from the four quadrants of the McGrath Task Circumplex (McGrath, 1984), an established and validated taxonomy characterizing tasks according to the dominant coordination process required for its accomplishment by a group, and included brainstorming, solving visual puzzles, making moral judgments, negotiating over limited resources, etc. See the Supplementary Appendix for a description of all tasks used. The first principal component derived from performance on all tasks served as the measure for collective intelligence. The Internal reliability (Cronbach’s α) for the collective intelligence measure was 0.72.

*Team learning* was calculated as the rate of change (or slope) in earnings for each group across the ten rounds of the game.

## Results

Descriptive statistics and intercorrelations are presented in Table 1.

**Table 1:** Team means and intercorrelations between cognitive style diversity, collective intelligence, and learning.

		1	2	3	4	5
1	Cognitive style level	–				
2	Cognitive style diversity	0.06	–			
3	Collective intelligence	0.09	−0.06	–		
4	Initial performance	−0.24^∗^	0.04	0.03	–	
5	Team learning	0.24^∗^	−0.05	0.20^∗^	−0.58^∗∗^	–
	Mean	434.35	20.15	0.14	2660.20	31.79
	*SD*	17.03	6.38	1.02	1090.90	84.37
	Minimum	395.03	4.95	−3.20	120	−167.27
	Maximum	480.61	33.30	3.31	4000	264.68

*Correlations are controlled for team size. ^∗^p < 0.05; ^∗∗^*p* < 0.01 (Two-tailed).*

**FIGURE 2:**
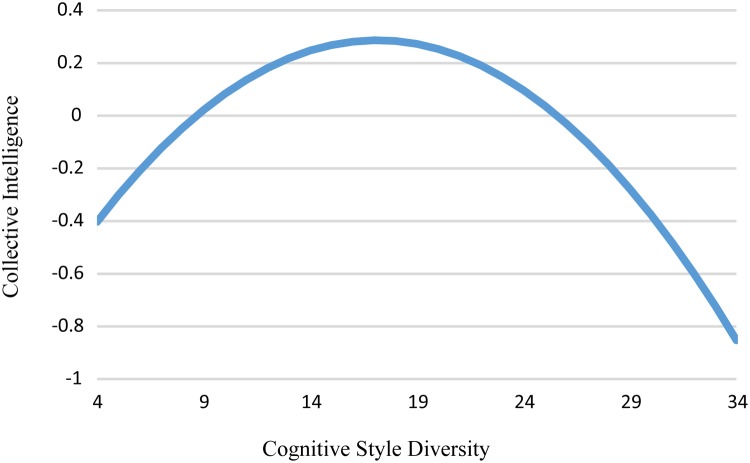
The relationship between cognitive style diversity and collective intelligence controlling for team size and cognitive style level.

Hypothesis 1, predicting an inverted U-shaped relationship between collective intelligence and cognitive diversity, was supported. The regression analysis, controlling for team size, and cognitive style level, demonstrated that the quadratic relationship between cognitive style diversity and collective intelligence was negative (indicating inverted U-shaped relationship) and significant: β = −0.91, *t* = −2.19, *p* = 0.03, *R^2^* = 0.40 (Table 2, Column 2; Figure 2).

**Table 2:** Results testing hypotheses 1 and 2 using OLS regression.

Dependent variable	Collective intelligence		Team learning	
	1	2	3	4
Team size	0.62^∗^	0.60^∗^	0.03	−0.15
Cognitive style level	0.07	0.06		
Cognitive style diversity	−0.06	0.85^∗^		
(Cognitive style diversity) ^2^		−0.91^∗^		
Learning intercept			−0.52^∗∗^	−0.54^∗∗^
Collective intelligence				0.29^∗∗^

*R*^2^	0.37	0.40	0.28	0.34
F	18.04^∗∗^	15.28^∗∗^	18.68^∗∗^	15.81^∗∗^
DeltaR^2^		0.03^∗^		0.06^∗∗^

*^∗^p < 0.05; ^∗∗^p < 0.01 (Two-tailed).*

Hypothesis 2 predicted a positive linear relationship between the team’s collective intelligence and team learning. A slope and intercept of the performance on the ten rounds were extracted for each team using OLS. The slope was used as the measure for learning. We found that collective intelligence positively and significantly related to team learning: β = 0.29, *t* = 2.74, *p* = 0.007, *R^2^* = 0.34, controlling for the intercept and team size (Table 2, Column 4). Additionally, we tested for this prediction using latent growth curve modeling, and also found evidence for the hypothesis; β = 0.022, *z* = 3.03, *p* < 0.05, controlling for initial choice and team size.

**FIGURE 3:**
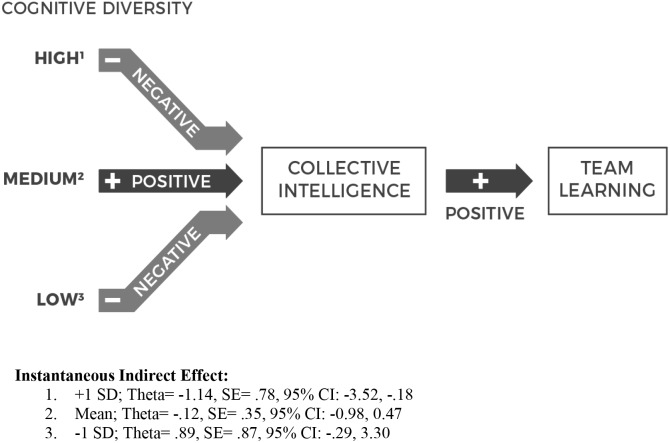
The relationship between cognitive style diversity, collective intelligence and team learning.

Hypothesis 3 predicted that there will be an indirect relationship between cognitive style diversity and team learning, mediated by collective intelligence. Mediation and bootstrapping analyses with 5000 samples (medcurve; Hayes and Preacher, 2010) indicated that there was an indirect relationship between cognitive style diversity and team learning through collective intelligence. As expected, this indirect relationship was evident at high levels of cognitive style diversity (+1 SD); *O* = −1.14, controlling for team size, cognitive style level and the learning intercept. A bootstrap analysis revealed that the 95% bias-corrected confidence interval for the size of the indirect effect excluded zero (−3.52, −0.18). This result suggests that high levels of cognitive style diversity curb team learning indirectly by reducing collective intelligence (Figure 3).

## Discussion

This research highlights the implications of cognitive diversity for longer-term organizational outcomes such as team learning. By synthesizing existing perspectives on diversity, particularly the delicate balance between the informational and signaling benefits and detrimental coordination effects of increased diversity, the study showed that there is an inverted-U-shaped relationship between cognitive style diversity and team collective intelligence; studying such non- monotonic relationships has also been suggested by researchers to move teams research forward (Van der Vegt and Bunderson, 2005). Additionally, moving away from the main effects approach (van Knippenberg and Schippers, 2007), this study demonstrates that cognitive style diversity indirectly influences team implicit learning through collective intelligence; specifically, at high levels of cognitive style diversity, collective intelligence is reduced, which then lowers the rate at which the team learns. Also, by investigating team learning in the form of growth trajectory of teams, this research subscribes to a dynamic approach to studying teams (Ilgen, 1999; McGrath Joseph et al., 2000). Below we discuss some theoretical and practical implications of this research, along with ideas for future research.

### Theoretical Implications

This study highlights the importance of team inputs in determining how well a team performs on a wide array of tasks consistently, and on team learning. As highlighted in the law of requisite variety, in order to tackle an array of problems from different domains, a team needs to possess a set of perspectives or skills that match the array of domains from which the tasks are drawn. We studied cognitive styles as team inputs. Understanding the connections between new, task-oriented forms of diversity and team states, processes and outcomes has been consistently recommended by researchers to move research in the team diversity area forward (Mannix and Neale, 2005; van Knippenberg and Schippers, 2007; Mohammed and Harrison, 2013). We answer this call, by going beyond characteristics that serve as proxies and focus directly on the differences in how individuals organize and process information (Kozhevnikov, 2007; Martinsen et al., 2011; Kozhevnikov et al., 2014) as an indicator of the different task-related perspectives and skills that exist within diverse teams.

By studying new types of task-relevant diversity variables, we accordingly start exploring team states and processes that may have been less relevant to other types of diversity variables, and hence understudied in the field. We theorize that having cognitive style diversity is likely to ensure that the team has access to different ways of approaching the problem at hand. It can also reduce the chances that the team will fall into confirmation bias traps where members confirm each other’s beliefs as a result of viewing information similarly. Cognitively diverse teams may more easily catch each other’s blind spots, which would facilitate performance across domains. While having the maximum amount of perspectives possible is likely to give the team the best set of resources to approach an array of problems, it is important to take coordination processes into account, which may be influenced by how team inputs are configured in the team. As indicated by Steiner (1972), when coining the term process loss, teams often perform suboptimally given the member resources because of coordination and communication difficulties. Therefore, as supported by our results, a moderate level of diversity appears to provide the requisite level of cognitive resource without being completely offset by the associated process losses, and influences the team’s collective intelligence.

Furthermore, we find that a team’s collective intelligence influences how it improves in implicit coordination as a function of experience. As demonstrated by the analyses supporting Hypothesis 2, we find that collective intelligence is positively related to team learning, characterized here by the rate of change in its implicit coordination. Because coordination is a dynamic process, it is important to understand not only how it occurs at one point in time, but also how it changes in teams as they gain experience, which this study was designed to investigate. Interestingly, we also find that collective intelligence in the team is not significantly correlated with initial levels of coordination, as seen in the score in the first round. Hence, teams with high levels of collective intelligence did not start out with an advantage in the tacit coordination game, which would have been an alternative explanation for higher rates of learning. Researchers have posited that information on initial team performance can be seen as a form of feedback (Schippers et al., 2013), and that feedback should inform subsequent group processes and performance (Peterson and Behfar, 2003). Further, we argue that not all teams will be equally skilled at making use of this feedback. We contend that teams with high levels of collective intelligence will use this feedback to their advantage, showing greater improvement with experience.

We found that collective intelligence was a mechanism through which cognitive style diversity indirectly influenced team learning. We found that this indirect effect existed when teams were high in cognitive style diversity. This indicates that high levels of cognitive style diversity interfere with the rate at which the team improves in its coordination by reducing the team’s collective intelligence. Since high levels of cognitive style diversity are more likely to hurt team coordination processes, when compared to low levels, it is logical that this mediation is supported only at high levels of diversity.

These findings have several implications for teams research broadly. Most research investigates the impact of group inputs and/or processes on performance at one given time. While that stream of research has important implications since some organizational groups may indeed be assembled to perform one task, it does not inform us about the way in which the group is likely to evolve over time (Marks et al., 2001), which is important in understanding teams as complex, adaptive, dynamic systems that are embedded in constantly changing contexts (Ilgen, 1999; McGrath Joseph et al., 2000).

### Practical Implications

There is increasing recognition that effective collaboration is crucial for an organization’s competitive advantage, and further increasingly greater numbers of employees are employed in the domain of knowledge work (Gibson, 2001; Nonaka, 2008). These findings have important implications for how we might enable team-based outcomes through understanding the implications of cognitive diversity on knowledge work in organizations. For instance, it will be important to investigate further how to compose teams to achieve moderate levels of diversity, possibly by combining some team members who are similar to one another with others who are different. Recent work on cultural brokerage (Jang, 2017) and cognitive versatility (Aggarwal and Molinaro, 2013) suggests that embedding individuals with relevant intrapersonal diversity may help bridge the divide between otherwise large differences among members. Such approaches may enable organizations to realize the benefits while avoiding the costs of cognitive diversity in teams. By focusing on a team’s collective intelligence, managers can make better assessments about which teams are likely to consistently perform well across different tasks; additionally, by focusing on team learning managers can adopt a sustainable far-sighted, rather than myopic, understanding of how teams are likely to evolve over time.

### Limitations and Future Directions

We studied groups in a lab setting, which allows performance to be more directly measured at multiple time points, and can enable better control over other potential sources of variance in the group, task, or learning environment (Argote, 1993). However, this relationship needs to be further examined in field settings. The link between diversity and performance has been shown to be similar in lab and field settings, though, leading us to anticipate similar results in field settings (Hoever et al., 2012; van Dijk et al., 2012).

The study was also designed to adjust for biases common in behavioral research. For example, common method bias, relevant when different constructs are measured with the same method, threatens validity since at least some of the observed covariation among measures may be due to the fact that they share the same method of measurement (Podsakoff et al., 2012). We mitigated this by having different modes of measuring the different constructs, which has been recommended as a procedural remedy for this bias, and the inclusion of both self-reported measures (i.e., cognitive styles), and objective measures (i.e., collective intelligence and team learning). Further, another way to control for this bias is to introduce a separation between the measures of the predictor and criterion variables such as temporal separation i.e., a time delay between measures (Feldman and Lynch, 1988; Podsakoff et al., 2003). The longitudinal design of the study also helps control for it. We increased internal validity by randomly assigning individuals to teams (rather than introducing error by self-selection). External validity is also increased due to the use of general population, rather than a student-only sample.

We have several recommendations for future research. In the current research, we focused on learning by doing. Future research should also explore collective intelligence in the context of vicarious learning. As posited by scholars, importing knowledge from other groups is a powerful mechanism for group learning (Argote et al., 2001). Cognitive, motivational, and social factors all affect the ease of transferring knowledge across groups. An unanswered research question pertains to the link between cognitive style diversity, collective intelligence and vicarious learning. Further, explicit knowledge is more likely to be learned vicariously than tacit knowledge. It could be interesting study the implicit versus explicit dimensions of learning in conjunction with the direct versus vicarious modes of learning. Since collective intelligence influences implicit learning in a team, it could be that teams with higher collective intelligence are also able to learn both explicit and tacit knowledge vicariously more effectively than teams with lower collective intelligence. This is a fruitful avenue for future research.

Future research could also explore the moderators to these relationships. In this study, we randomly assign participants to teams of different sizes, and controlled for team size in the analysis, for a more robust test of the hypotheses. However, an alternative design could test the role of team size as a moderator to the relationship between team cognitive diversity and collective intelligence. Another valid moderator influencing the relationship between collective intelligence and team learning could be team reflexivity or the conscious reflection on team functioning (Schippers et al., 2013)^1^. Team reflexivity has been shown positively influence team learning and future performance of teams that perform at low levels initially, as compared to teams that perform well initially. Based on this research (Schippers et al., 2013), one prediction would be that teams with low levels of collective intelligence would benefit from team reflexivity in improving future team outcomes including team learning. But, since team reflexivity comes at a cost of consuming cognitive resources, it might be that teams with high levels of collective intelligence might not benefit from it, or even be hurt by it. Since it might not be always viable to change team composition in organizations, it could be that interventions related to team reflexivity could help teams with low levels of collective intelligence to have increased learning. These questions are fruitful avenues for future research.

## Conclusion

Understanding how teams cope effectively with changing environments is an important question for organizational research. Teams that have the ability to perform effectively across changing contexts, and align their member resources into processes that yield consistency in performance, are likely to be more beneficial for organizations than teams that falter when facing a change in the environment. This study shows that having a moderate amount of cognitive style diversity facilitates such team ability since having too little is unlikely to provide teams with the cognitive capacity and flexibility to tackle tasks that require different ways of encoding and processing information, while having too much is likely to disrupt coordination in teams. Further, such team ability, or collective intelligence, predicts the rate at which teams improve in their implicit coordination, a process that is extremely important in high reliability organizations, among others. This study also demonstrates that cognitive style diversity indirectly influences the rate at which the team improves its implicit coordination over time through its collective intelligence. Overall, this research identifies a set of important cognitive inputs, states, and processes that further our understanding of teams in a dynamic way.

## Author Contributions

All authors developed the study concept, contributed to the study design, and participated in the interpretation of analyses. Testing, data collection, and data analyses were performed and also manuscript was drafted by IA and AWW. CFC and TWM provided critical revisions.

## Conflict of Interest Statement

The authors declare that the research was conducted in the absence of any commercial or financial relationships that could be construed as a potential conflict of interest.

## Footnotes

1 We thank reviewers for this suggestion.
