# Supplementary material for: The Impact of Cognitive Style Diversity on Implicit Learning in Teams
Source: Front Psychol. 2019 Feb 7;10:112. doi: 10.3389/fpsyg.2019.00112 (PMC6374291; doi:10.3389/fpsyg.2019.00112)
Supplement: Supplementary file 1 [file Table_1.DOCX]

**Supplementary Methods Appendix**

**Collective Intelligence (CI) Measurement**

We selected tasks from the McGrath Task Circumplex (Spearman, 1904), an established and validated taxonomy characterizing tasks according to the dominant coordination process required for its accomplishment by a group. The Taxonomy identifies four main types of tasks: (1) Quadrant I includes “Generate” tasks which include brainstorming tasks and anything involving the development of new ideas or information; (2) Quadrant II includes “Choose” tasks which involve deciding about issues that either have a correct answer or which are matters of judgment, with some research noting important distinctions among intellective and judgmental tasks (*2*); (3) Quadrant III includes “Negotiate” tasks which involve resolving conflicts of interest or points of view; and (4) Quadrant IV includes “Execute” tasks which involve performances and psycho-motor tasks. We included at least one task from each quadrant. The tasks are described below.  

*Brainstorming(Quadrant I).* Groups spent 10 minutes brainstorming possible uses for a brick. Groups received one point for each non-redundant idea they generated, independent of quality of the ideas.

*Group Matrix Reasoning (Quadrant II).* Groups completed the even-numbered questions of RAPM questions as a group. Groups were scored on the number of items answered correctly.

*Group Moral Reasoning* *(Quadrant II)****.*** Using the “Disciplinary Action Case” (*3*), groups decided on disciplinary actions in a fictitious case in which a college basketball player bribed an instructor to change his grade on an exam. The groups were given a list of five issues having to do with how to treat the athlete and the instructor. Issues included what to do about the student’s grade in the course and whether to suspend him from school. The groups' task was to select one choice from a list of alternatives provided for each issue. In addition, groups were told to take into account the conflicting interests of the faculty, college administration, and the athletic department when making their decisions. Responses were scored using a rubric that reflected the degree to which the groups considered the balance of competing perspectives on the problem.

*Plan shopping trip (Quadrant III)*.  The groups' task was to plan a shopping trip as if they were all residents of the same house sharing a single car. Each group member was given a different list of groceries they needed for the week, and various constraints applied.  For instance, there were better and worse places to buy the different items, with cheaper and higher quality options requiring more driving time. Maps were provided with information on distances and time for reaching each store. Solutions were scored as follows: (a) each item purchased = +1 point, (b) bonus for high quality item = +1 point, (c) bonus for lower priced alternative = +2 points, (d) penalty for leaving frozen items in the car beyond 30 minutes = loss of all points for that item.   The groups' goal was to work out a plan in which they could purchase as many high-value items from each of their lists as possible and thus earn as many points as possible for their group.

*Group typing (Quadrant IV).* Groups were provided a hard copy of a complicated text and worked for 10 minutes to simultaneously type as much of the text as possible into a shared online document.  Participants were each seated in front of a separate computer and worked in the shared online document where they could see each others' work with a slight delay.  Teams earned one point for each word correctly typed, and lost one point for each word skipped and for each typo. Team members thus needed to carefully coordinate their work to avoid typing over the work of other members or skipping whole sections (which would result in the loss of many points).

The first principal component derived from performance on all tasks served as the measure for collective intelligence. The first principal component accounted for 44% of the variance; the internal reliability (Cronbach’s α) for the collective intelligence measure was 0.72.
